# Two Surgical Cases of Combined Hepatocellular-Cholangiocarcinoma, Intermediate-Cell Subtype: Potentially Characteristic Gross Features

**DOI:** 10.1155/2018/8423939

**Published:** 2018-06-05

**Authors:** Jiro Watanabe, Sohsuke Yamada, Yasuyuki Sasaguri, Masaru Inagaki, Hiromi Iwagaki

**Affiliations:** ^1^Laboratory of Pathology, National Hospital Organization, Fukuyama Medical Center, Fukuyama, Japan; ^2^Department of Pathology and Laboratory Medicine, Kanazawa Medical University, 1-1 Uchinada, Ishikawa 920-0293, Japan; ^3^Laboratory of Pathology, Fukuoka Tokushukai Hospital, Fukuoka, Japan; ^4^Department of Surgery, National Hospital Organization, Fukuyama Medical Center, Fukuyama, Japan

## Abstract

We herein reported two rare surgical cases of primary combined hepatocellular-cholangiocellular carcinoma, intermediate-cell subtype (CHC-INT), showing potentially characteristic and specific gross findings on their cut surface: both CHC-INTs demonstrated poorly demarcated and expansive and/or infiltrative hepatic nodules in lobulated margins, appearing clearly whitish in color. We were finally able to accurately diagnose the current lesions after thorough analyses including an appropriate and wide panel of immunohistochemical antibodies. Despite that, all pathologists should be aware that the potentially characteristic gross features of primary CHC-INT might also be one of the powerful supplementary tools for reaching its correct, conclusive diagnosis.

## 1. Introduction

In 1903, Wells first reported the features of primary malignant liver tumor, combined hepatocellular- (HCC-) cholangiocellular (CCC) carcinoma (CHC), and following that, Allen and Lisa, Goodman et al., and Taguchi et al. subclassified CHC variously [1–3]. Recently, in the latest World Health Organization (WHO) classification of the digestive system, CHC is ultimately divided into (1) classical type and (2) subtypes with stem cell features [4]. Furthermore, the latter (2) has been subdivided into (i) typical subtype, (ii) intermediate-cell subtype (CHC-INT), and (iii) cholangiolocellular subtype [4]. In particular, the histopathological findings of rare (ii) CHC-INT characteristically demonstrate a proliferation of relatively small and uniform carcinoma cells having intermediate features between hepatocytes and cholangiocytes, i.e., HCC and CCC, arranged in strands, solid nests, trabeculae, and/or ill-defined gland-/tubule-like structures [4]. A substantial number of interesting papers focusing especially on the histopathological and immunohistochemical features of CHC-INT were published [3, 5]; however, within our thorough investigation, there has been no detailed description regarding the gross findings reported in the English literature. Indeed, the above WHO classification has merely stated that the gross morphology of CHC-INT is not significantly different from that of HCC [4]; however, we cannot completely agree with that description. We herein briefly report two rare surgical cases of primary CHC-INT, showing potentially characteristic and specific gross features on their cut surface.

## 2. Case Presentation (Case 1)

**Figure 1:**
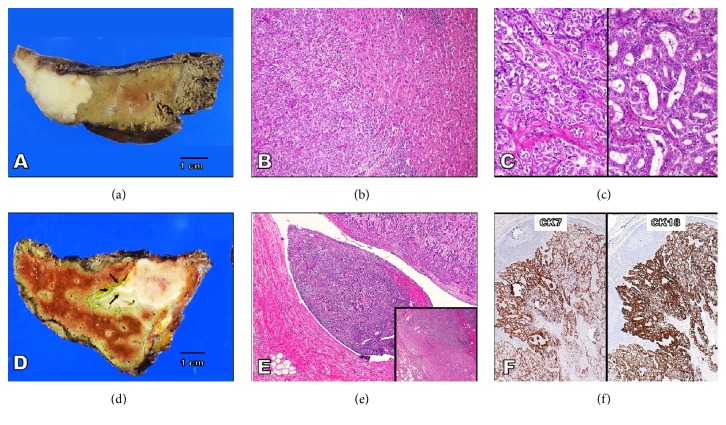
**The gross, microscopic, and immunohistochemical examinations of the primary CHC-INT**. (a) The cut surface of the first CHC-INT case characteristically shows a poorly demarcated peripheral nodule in lobulated margins, measuring 32 x 21 mm in diameter, which appears clearly whitish in color. Bar = 1 cm. (b) A microscopic examination of the first CHC-INT case (H&E staining) demonstrates an unencapsulated, ill-defined, and expansive nodule (left side), arranged predominantly in a proliferation of solid nests pattern (left side). (c) The first CHC-INT (H&E staining) shows a solid proliferation of atypical epithelial cells, forming trabeculae (left panel) and/or ill-defined, fused tubule-like structures (right panel). These atypical cells are small-to-medium-sized and relatively uniform, having enlarged hyperchromatic nuclei and scant cytoplasm. (d) The cut surface of the second CHC-INT case characteristically reveals a poorly demarcated portal nodule in lobulated margins with central necrosis, measuring 35 x 26 mm in diameter, which appears clearly whitish in color, accompanied by portal vein permeation (arrows). Bar = 1 cm. (e) A microscopic examination of the second unencapsulated CHC-INT tumor (H&E staining) shows a solid proliferation of atypical epithelial cells, arranged predominantly in solid nests, trabeculae, and/or irregular and fused tubule-like structures, aggressively involving the portal vein (right upper side to center) with focal perineural invasion (inset). Left lower side is the intact wall of the portal vein. (f) Immunohistochemistry demonstrates that those second CHC-INT carcinoma cells are specifically positive for not only CK7 (left; cholangiocytes marker) but also CK18 (marker for both hepatocytes and cholangiocytes).

The first patient, who was a man in his late seventies with an unremarkable previous medical history, presented with isovascular nodule accompanied by slow venous wash-out on abdominal dynamic CT in the left lobe of liver. The laboratory data, including the blood cell count, chemistry, and tumor marker levels, were within the normal limits, with the exception of mildly elevated CRP (0.39 mg/dL) and decreased hemoglobin (8.9 g/dL) levels. Neither infection of HBV nor infection of HCV was noted. Based on the clinical findings, the initial diagnosis by the clinicians was most likely HCC, and, thus, left partial hepatectomy was performed. On gross examination, the cut surface of hepatic nodule (Figure 1(a)) showed a poorly demarcated peripheral nodule in lobulated margins, measuring 32 x 21 mm in diameter, which appeared clearly whitish in color. The background of this liver showed no remarkable change (Figure 1(a)). A microscopic examination of the tumor demonstrated an unencapsulated, ill-defined, and expansive nodule (Figure 1(b)). This cancerous nodule showed a solid proliferation of atypical epithelial cells, arranged predominantly in solid nests (Figure 1(b)), trabeculae, and/or ill-defined, fused tubule-like structures (Figure 1(c)). On a high-power view, these atypical cells were small-to-medium-sized and relatively uniform, having enlarged hyperchromatic nuclei and scant cytoplasm without any evidence of intracytoplasmic mucin (Figure 1(c)). Intriguingly, prominent fibrous stroma was not evident in this tumor. Immunohistochemistry revealed that the abovementioned carcinoma cells were specifically positive for not only CK7 (cholangiocytes marker) but also CK8/CK18 (markers for both hepatocytes and cholangiocytes) and CK19/CD56 (potential stem cells markers), whereas they were negative for Hepatocyte (hepatocytes marker) [3, 5]. Based on all of these features, the final diagnosis was primary CHC-INT. To date, this patient has been followed up for 1 year since surgery, and he remains well without any sign of recurrence.

## 3. Case Presentation (Case 2)

The second patient, who was also a man in his early seventies with more than 25-year follow-up for HCV-positive chronic hepatitis and recurrent HCC, presented with mildly hypervascular and ring-enhanced nodule accompanied by venous wash-out on abdominal dynamic CT in the S5 of the remnant liver. The laboratory data were mostly within the normal limits, with the exception of mildly elevated AST (37 IU/L), total bilirubin (1.8 mg/dL), and CEA (5.55 ng/mL) levels. No infection of HBV was observed. Based on the clinical findings, the initial diagnosis by the clinicians was recurrent HCC, and, thus, partial S5 hepatectomy was performed. On gross examination, the cut surface of hepatic nodule (Figure 1(d)) showed a poorly demarcated portal nodule in lobulated margins with central necrosis, measuring 35 x 26 mm in diameter, which appeared clearly whitish in color, accompanied by not only gross but also histopathological portal vein permeation (Figure 1(e)). A microscopic examination of the tumor showed an unencapsulated, ill-defined, and expansive/infiltrative nodule, displaying a solid proliferation of atypical epithelial cells, arranged predominantly in solid nests, trabeculae, and/or irregular and fused tubule-like structures, aggressively involving the portal vein with focal perineural invasion (Figure 1(e)). On a high-power view, these atypical cells were very similar to those of the abovementioned first case. Prominent fibrous stroma was not seen either. The background of this liver showed mild chronic hepatitis (F1/A1) and steatosis. Immunohistochemistry showed that those carcinoma cells were specifically positive for not only CK7 (Figure 1(f)) but also CK18 (Figure 1(f)) and c-kit (potential stem cells marker) [3, 5], whereas they were negative for CK8, Hepatocyte, and CD56. Based on all of these features, the final diagnosis was primary CHC-INT as well. The recurrence of CHC-INT in the remnant liver occurred 1 year and 3 months after this surgery, but he remains not worse with follow-up for the postoperative 2 years.

## 4. Discussion

It is very likely that the current report of two surgical CHC-INT patients is clinicopathologically remarkable for one reason at least. In case of the present gross findings for poorly demarcated, unencapsulated, and lobulated nodules looking clearly whitish in color on the cut surface, we pathologists should consider the rare possibility of CHC-INT, rather than common HCC. In addition, a wide panel of immunohistochemical analyses should be critically performed, as shown here. In our opinion, the macroscopic features of HCCs appear yellow-whitish, tan to green, but not clearly whitish, in color, even though further modified by varying degrees of necrosis/hemorrhage and the production of bile or fat. In fact, the most critical differential diagnosis in the present cases was HCC, despite the fact that it should be relatively easy to rule out this possibility through a clinicopathologic examination or immunohistochemistry. Since most CHCs are known to generally have a worse outcome, growing rapidly and showing aggressive/infiltrative behaviours [3, 4, 6], as in our second case, alerting the surgeons to the postoperative careful follow-up and additional treatment, at the very least, should be raised. Furthermore, Sasaki et al. have recently reported that, among the subtypes with stem cell features of CHC, CHC-INT is significantly associated with bigger tumor size or higher histological grade of coexistent HCC [7]; however, its prognosis after curative surgical resection is unknown, with conflicting evidence based on small series and patients' numbers [3, 4, 6, 7]. Nevertheless, it would be intriguing to assess the significance of those unique gross features and prognoses on future larger studies. This short case report, taken together with the potentially specific findings of cut surface for CHC-INT, might promote interest within the scientific community.

## 5. Conclusion

In conclusion, we herein reported two rare surgical cases of primary CHC-INT, showing potentially characteristic and specific gross findings on their cut surface: both CHC-INTs demonstrated poorly demarcated and expansive and/or infiltrative hepatic nodules in lobulated margins, appearing clearly whitish in color. We were finally able to accurately diagnose the current lesions after thorough analyses including an appropriate and wide panel of immunohistochemical antibodies. Despite that, all pathologists should be aware that the potentially characteristic gross features of primary CHC-INT might also be one of the powerful supplementary tools for reaching its correct, conclusive diagnosis.

## Data Availability

The dataset supporting the findings and conclusions of this report is included within the article.

## Abbreviations

CT: Computed tomography
HBV: Hepatitis type B virus
HCV: Hepatitis type C virus
CK: Cytokeratin
Hepatocyte: Staining for hepatocyte antigen
AST: Aspartate aminotransferase
CEA: Carcinoembryonic antigen
F1/A1: Fibrosis staging 1/activity grading 1.
